# Prognostic nomogram to predict the overall survival of patients with early-onset colorectal cancer: a population-based analysis

**DOI:** 10.1007/s00384-021-03992-w

**Published:** 2021-07-29

**Authors:** Junxian Wu, Linbin Lu, Hong Chen, Yihong Lin, Huanlin Zhang, Enlin Chen, Weiwei Lin, Jie Li, Xi Chen

**Affiliations:** Department of Oncology, The 900th Hospital of the People’s Liberation Army Joint Service Support Force, Fuzong Clinical Medical College of Fujian Medical University, Fuzhou, China

**Keywords:** Nomogram, Early-onset colorectal cancer, SEER, Prediction, Overall survival

## Abstract

**Purpose:**

The present study aimed to identify independent clinicopathological and socio-economic prognostic factors associated with overall survival of early-onset colorectal cancer (EO-CRC) patients and then establish and validate a prognostic nomogram for patients with EO-CRC.

**Methods:**

Eligible patients with EO-CRC diagnosed from 2010 to 2017 were extracted from the Surveillance, Epidemiology, and End Results (SEER) database. Patients were randomly divided into a training cohort and a testing cohort. Independent prognostic factors were obtained using univariate and multivariate Cox analyses and were used to establish a nomogram for predicting 3- and 5-year overall survival (OS). The discriminative ability and calibration of the nomogram were assessed using C-index values, AUC values, and calibration plots.

**Results:**

In total, 5585 patients with EO-CRC were involved in the study. Based on the univariate and multivariate analyses, 15 independent prognostic factors were assembled into the nomogram to predict 3- and 5-year OS. The nomogram showed favorable discriminatory ability as indicated by the C-index (0.840, 95% CI 0.827–0.850), and the 3- and 5-year AUC values (0.868 and 0.84869 respectively). Calibration plots indicated optimal agreement between the nomogram-predicted survival and the actual observed survival. The results remained reproducible in the testing cohort. The C-index of the nomogram was higher than that of the TNM staging system (0.840 vs 0.804, *P* < 0.001).

**Conclusion:**

A novel prognostic nomogram for EO-CRC patients based on independent clinicopathological and socio-economic factors was developed, which was superior to the TNM staging system. The nomogram could facilitate postoperative individual prognosis prediction and clinical decision-making.

## Introduction

Colorectal cancer (CRC) ranks the third most common cancer worldwide (10.2%) but second in terms of mortality (9.2%) when men and women are combined [1]. It is noteworthy that the incidence of early-onset CRC (EO-CRC, aged < 50 years) patients increased by approximately 2% annually since the mid-1990s, compared to the decreasing incidence in older populations [2, 3] in many regions across the globe [4–6]. It was projected that, by 2030, 10.9% of colon and 22.9% of rectal cancers would be diagnosed in patients younger than 50 years [7]. So, it is necessary to found crucial prognostic factors for predicting the survival outcome of EO-CRC patients, which is beneficial to further clinical decision-making.

Currently, the American Joint Committee on Cancer (AJCC) TNM staging system is widely used for prognosis prediction and medical treatments in many cancers. However, the TNM staging does not deal with all survival discrepancies. For example, some colon cancer patients in stage III had a statistically better prognosis than those with stage IIB and IIC according to this staging system [8]. Furthermore, many other clinicopathological factors, such as primary site, tumor size, lymph node ratio (LNR), pretreatment carcinoembryonic antigen (CEA) level, circumferential resection margin (CRM), and tumor deposits, have been demonstrated to influence the survival outcome in colorectal cancer, while they were not sufficiently utilized by the TNM staging system. Therefore, in clinical practice, an integrated prognostic judgment system incorporating crucial factors is needed.

Nomograms are statistical predictive models that incorporate independent factors of prognosis to estimate prognosis for individual patients. They have been built for various types of cancers [9–13] and have shown advantages over the TNM staging system [10, 11, 14, 15]. However, nomograms regarding EO-CRC patients are still rare nowadays.

Therefore, the present study aimed to identify clinicopathological and socio-economic prognostic factors associated with overall survival of EO-CRC patients using a large multi-institutional data from the Surveillance, Epidemiology, and End Results (SEER) database, then to establish and internally validate a nomogram for predicting the 3- and 5-year OS of EO-CRC patients.

## Methods

### Data source and patient selection

**Fig. 1:**
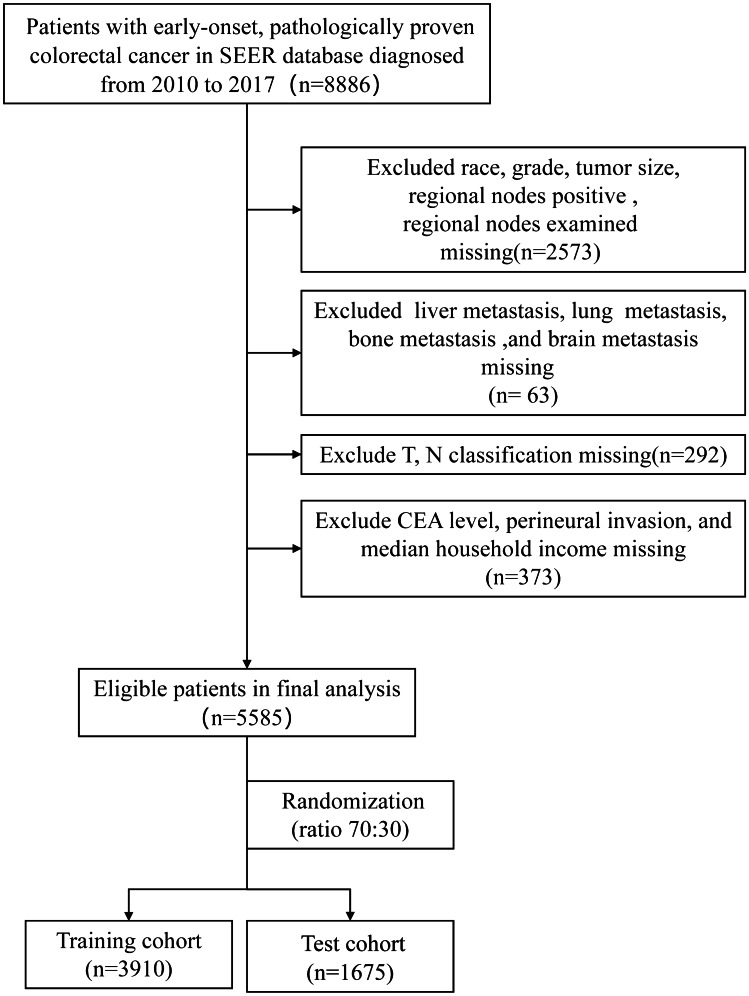
The workflow of the patient selection process

The SEER program of the National Cancer Institute (NCI) collects information on cancer incidence and survival from 17 population-based cancer registries and represents about 28% of the US population. In this study, a total of 8886 pathologically proven EO-CRC patients who were diagnosed from January 1, 2010, to December 31, 2017, were retrospectively extracted from the SEER database using the SEER*Stat program (v 8.3.6). Patients with EO-CRC were identified by the ICD-O-3 site code (C18.0, C18.2, C18.3, C18.4, C18.5, C18.6, C18.7, C19.9, C20.9) and the cancer staging scheme (version 0204). The inclusion criteria were as follows: (1) patients were 15–50 years old, (2) CRC was the only primary cancer, (3) complete survival information, and (4) follow-up > 1 month. Patients who had missing or incomplete clinicopathological and socio-economic information (primary site, histological type, grade, tumor size, regional nodes examined, metastatic situation, tumor stage, CEA level, perineural invasion, median household income) were excluded from this study. The detailed patient selection workflow is shown in Fig. 1. Eligible patients were randomly divided into a training cohort and a testing cohort (ratio, 70:30). The training cohort was used to explore the prognostic factors, and to construct a nomogram, the testing cohort was used for internal validation of the nomogram. This study was conducted under the SEER data use agreement, and patient informed consent was not required given the anonymized, de-identified data in the SEER database.

### Variables and outcome

Eighteen factors, including sex, race, primary site, histology, grade, tumor size, number of examined regional nodes, LNR, liver metastasis, lung metastasis, bone metastasis, brain metastasis, TNM stage, T stage, N stage, CEA, perineural invasion, and median household income, were retrieved to predict prognosis of the training cohort. The primary site was defined as right-side (cecum, ascending colon, hepatic flexure of colon, transverse colon), left-side (splenic flexure of colon, descending colon, sigmoid colon, rectosigmoid), and rectum. The LNR was calculated by dividing the metastatic node number by the examined regional node number. Overall survival (OS), the primary endpoint, was defined as the interval from diagnosis until death or last follow-up.

### Statistical analysis

Categorical variables were reported as whole numbers and proportions. The overall survivals of the study cohort were produced using the Kaplan–Meier method, and differences between overall survivals were examined using the log-rank test. The associations between clinicopathological, socio-economic variables and survival were evaluated using Cox proportional hazards regression models. Hazard ratios (HRs) were displayed with 95% CIs. Significant variables in the univariate analysis were subjected to multivariate Cox regression analysis by Backward stepwise selection under the Akaike information criterion (AIC). Variables statistically significant in the multivariate Cox regression analysis were determined as independent prognostic factors to predict the survival outcome. Then, these independent prognostic factors were used to establish a nomogram for predicting the 3- and 5-year OS of patients with EO-CRC. To allot points in the nomogram, the regression coefficients were used to define the linear predictor.

The performance of the nomogram was evaluated by the discriminatory ability and calibration [16]. The discriminatory ability refers to how well the model differentiates patients who will have an event from those who will not have an event. The concordance index (C-index) and the receiver operating characteristic (ROC) curve were applied to evaluate the discriminatory ability of our nomogram. A C-index or the area under the ROC curve (AUC) of 0.5 indicates the nomogram is devoid of discrimination, while a C-index or AUC of 1.0 suggests the perfect separation of patients with different results. A C-index or AUC more than 0.75 reflects useful discrimination [16]. The calibration refers to the consistency between the nomogram-predicted survival and the actual observed survival. Calibration plots were used to evaluate the calibration of our nomogram. In a calibration plot, the actual OS is plotted on the y-axis, and the nomogram-predicted OS is plotted on the x-axis. A perfect prediction would fall on a 45-degree diagonal line. All the statistical analyses were performed using SPSS version 25 and R software version 3.3.0 (Vienna, Austria; www.r-project.org). Only a two-tailed *P* value of < 0.05 was considered statistically significant. This study has been reported in line with the TRIPOD statement [17].

## Results

### Clinicopathological and socio-economic characteristics and survival outcomes of EO-CRC patients

Data on a total of 5585 eligible patients with early-onset colorectal cancer diagnosed from 2010 to 2017 were retrospectively collected from the SEER database. Patients were randomly divided into a training cohort (3910 patients) and a testing cohort (1675 patients). Clinicopathological and socio-economic characteristics of early-onset colorectal cancer patients are listed in Table 1.

**Table 1:** Clinicopathological and socio-economic characteristics of early-onset colorectal cancer patients from 2010 to 2017

Characteristics	All patients (*n* = 5585)	Training cohort (*n* = 3910)	Testing cohort (*n* = 1675)
	No. (%)	No. (%)	No. (%)
Sex			
Male	2996 (53.64)	2107 (53.89)	889 (53.07)
Female	2589 (46.36)	1803 (46.11)	786 (46.93)
Race			
White	3990( 71.44)	2780 (71.1)	1210 (72.24)
Black	817 (14.63)	578 (14.78)	239 (14.27)
Asian or Pacific Islander	696 (12.46)	500 (12.79)	196 (11.7)
American Indian/Alaska Native	82 (1.47)	52 (1.33)	30 (1.79)
Primary site			
Right-side colon	1548 (27.72)	1100 (28.13)	448 (26.75)
Left-side colon	2558 (45.8)	1790 (45.78)	768 (45.85)
Rectum	1479 (26.48)	1020 (26.09)	459 (27.4)
Histology			
Adenocarcinoma	5090 (91.14)	3565 (91.18)	1525 (91.04)
Non-adenocarcinoma	495 (8.86)	345 (8.82)	150 (8.96)
Grade			
Well	320 (5.73)	228 (5.83)	92 (5.49)
Moderate	4208 (75.34)	2953 (75.52)	1255 (74.93)
Poor	874 (15.65)	594 (15.19)	280 (16.72)
Undifferentiated	183 (3.28)	135 (3.45)	48 (2.87)
Tumor size			
<5 cm	2852 (51.07)	2026 (51.82)	826 (49.31)
≥5 cm	2733 (48.93)	1884 (48.18)	849 (50.69)
Regional nodes examined			
<12 nodes	869 (15.56)	611 (15.63)	258 (15.4)
≥12 nodes	4716 (84.44)	3299 (84.37)	1417 (84.6)
LNR			
0<=x<=0.2	4441 (79.52)	3115 (79.67)	1326 (79.16)
0.2<x<=0.6	895 (16.03)	626 (16.01)	269 (16.06)
x>0 6	249 (4.46)	169 (4.32)	80 (4.78)
Liver metastasis			
Yes	764 (13.68)	541 (13.84)	223 (13.31)
No	4821 (86.32)	3369 (86.16)	1452 (86.69)
Lung metastasis			
Yes	186 (3.33)	123 (3.15)	63 (3.76)
No	5399 (96.67)	3787 (96.85)	1612 (96.24)
Bone metastasis			
Yes	28 (0.5)	20 (0.51)	8 (0.48)
No	5557 (99.5)	3890 (99.49)	1667 (99.52)
Brain metastasis			
Yes	8 (0.14)	5 (0.13)	3 (0.18)
No	5577 (99.86)	3905 (99.87)	1672 (99.82)
Stage			
I	799 (14.31)	577 (14.76)	222 (13.25)
II	1283 (22.97)	893 (22.84)	390 (23.28)
III	2409 (43.13)	1668 (42.66)	741 (44.24)
IV	1094 (19.59)	772 (19.74)	322 (19.22)
T			
T1	542 (9.7)	403 (10.31)	139 (8.3)
T2	602 (10.78)	416 (10.64)	186 (11.1)
T3	3265 (58.46)	2307 (59)	958 (57.19)
T4	1176 (21.06)	784 (20.05)	392 (23.4)
N			
N0	2235 (40.02)	1582 (40.46)	653 (38.99)
N1	1919 (34.36)	1326 (33.91)	593 (35.4)
N2	1431 (25.62)	1002 (25.63)	429 (25.61)
CEA			
Positive	1602 (28.68)	1118 (28.59)	484 (28.9)
Negative	2217 (39.7)	1567 (40.08)	650 (38.81)
Unknown	1766 (31.62)	1225 (31.33)	541 (32.3)
Perineural invasion			
Yes	899 (16.1)	642 (16.42)	257 (15.34)
No	4257 (76.22)	2973 (76.04)	1284 (76.66)
Unknown	429 (7.68)	295 (7.54)	134 (8)
Median household income			
<50000 dollars	662 (11.85)	454 (11.61)	208 (12.42)
50000–75000 dollars	2830 (50.67)	1975 (50.51)	855 (51.04)
>75000 dollars	2093 (37.48)	1481 (37.88)	612 (36.54)

Most patients were male (53.64%) and White (71.44%), with a median household income level of 50,000–75,000 dollars (50.67%). The majority of patients had the adenocarcinoma histological type (91.14%), were moderately differentiated (75.34%), examined ≥ 12 regional nodes (84.44%), with LNR ranged from 0 to 0.2 (79.52%), without perineural invasion (76.22%). The left-side colon (45.8%) was the most common primary tumor site, followed by the right-side colon (27.72%), and rectum (26.48%). 51.07% of the patients developed a smaller tumor size (< 5 cm), while 48.93% of patients developed a larger tumor size (≥ 5 cm). Liver metastasis, lung metastasis, bone metastasis, and brain metastasis were observed in 13.68%, 3.33%, 0.5%, and 0.14% of the patients, respectively. Patients with TNM stage I, II, III, and IV tumors accounted for 14.31%, 22.97%, 43.13%, and 19.59% of all cases, respectively. In total, 28.68% of the patients were tested with positive pretreatment CEA, with the remaining patients having negative CEA (39.7%) or unknown CEA (31.62%).

At a median follow-up of 42.0 months (range from 1.0 to 95.0 months), 19.7% (772 of 3910) of the patients had died in the training cohort, and 20.6% (346 of 1675) of the patients had died in the testing cohort. The 3-year and 5-year overall survival were 80.7% (95% CI, 79.3–82.1%), and 72.5% (95% CI, 70.7–74.3%), respectively.

### Independent prognostic factors of early-onset colorectal cancer patients

Univariate Cox regression analysis indicated that race, primary site, histology, grade, tumor size, regional nodes examined, LNR, liver metastasis, lung metastasis, bone metastasis, brain metastasis, TNM stage, T stage, N stage, CEA, perineural invasion, and median household income were significantly associated with OS in the training cohort **(**Table 2).

**Table. 2:** Univariate cox regression analysis of overall survival in the training cohort

Characteristics	Hazard ratio	95% CI	*P*-value
Sex			
Male	Reference		
Female	1.14	0.99–1.31	0.075
Race			< 0.001
White	Reference		
Black	1.45	1.21–1.74	< 0.001
Asian or Pacific Islander	0.84	0.66–1.06	0.138
American Indian/ Alaska Native	2.25	1.40–3.59	0.001
Primary site			0.027
Right-side colon	Reference		
Left-side colon	0.82	0.70–0.97	0.019
Rectum	0.80	0.66–0.97	0.020
Histology			
Adenocarcinoma	Reference		
Non-adenocarcinoma	2.01	1.65–2.44	< 0.001
Grade			< 0.001
Well	Reference		
Moderate	1.13	0.80–1.60	0.481
Poor	2.68	1.86–3.85	< 0.001
Undifferentiated	4.22	2.78–6.43	< 0.001
Tumor size			
< 5 cm	Reference		
≥ 5 cm	1.62	1.41–1.87	< 0.001
Regional nodes examined			
< 12 nodes	Reference		
≥ 12 nodes	0.54	0.46–0.63	< 0.001
LNR			< 0.001
0 < = x < = 0.2	Reference		
0.2 < x < = 0.6	2.56	2.18–3.02	< 0.001
x > 0 6	7.12	5.75–8.82	< 0.001
Liver metastasis			
Yes	Reference		
No	0.14	0.13–0.17	< 0.001
Lung metastasis			
Yes	Reference		
No	0.18	0.14–0.23	< 0.001
Bone metastasis			
Yes	Reference		
No	0.08	0.05–0.14	< 0.001
Brain metastasis			
Yes	Reference		
No	0.18	0.04–0.72	0.016
Stage			< 0.001
I	Reference		
II	2.06	1.26–3.37	0.004
III	4.48	2.87–7.00	< 0.001
IV	26.64	17.17–41.33	< 0.001
T			< 0.001
T1	Reference		
T2	0.73	0.46–1.17	0.194
T3	1.83	1.32–2.55	< 0.001
T4	5.43	3.89–7.58	< 0.001
N			< 0.001
N0	Reference		
N1	2.43	1.99–2.98	< 0.001
N2	4.98	4.10–6.05	< 0.001
CEA			< 0.001
Positive	Reference		
Negative	0.29	0.25–0.35	< 0.001
Unknown	0.51	0.43–0.60	< 0.001
Perineural invasion			< 0.001
Yes	Reference		
No	0.34	0.29–0.40	< 0.001
Unknown	0.69	0.54–0.88	0.003
Median household income			0.003
< 50,000 dollars	Reference		
50,000–75,000 dollars	0.85	0.69–1.04	0.113
> 75,000 dollars	0.69	0.55–0.87	0.001

**Fig. 2:**
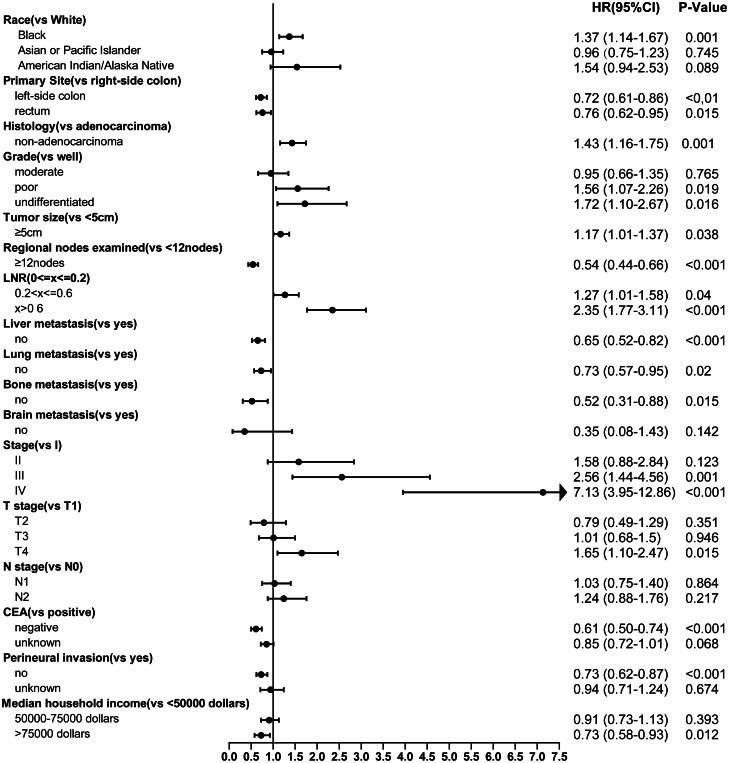
Multivariate cox regression analysis of overall survival in the training cohort. LNR, lymph node ratio; CEA, carcinoembryonic antigen

After controlling confounding factors, the multivariate Cox regression analysis demonstrated that race, primary site, histology, grade, tumor size, regional nodes examined, LNR, liver metastasis, lung metastasis, bone metastasis, TNM stage, T stage, CEA, perineural invasion, and median household income were independent prognostic factors of EO-CRC patients as shown in Fig. 2.

### Construction of the prognostic nomogram

**Fig. 3:**
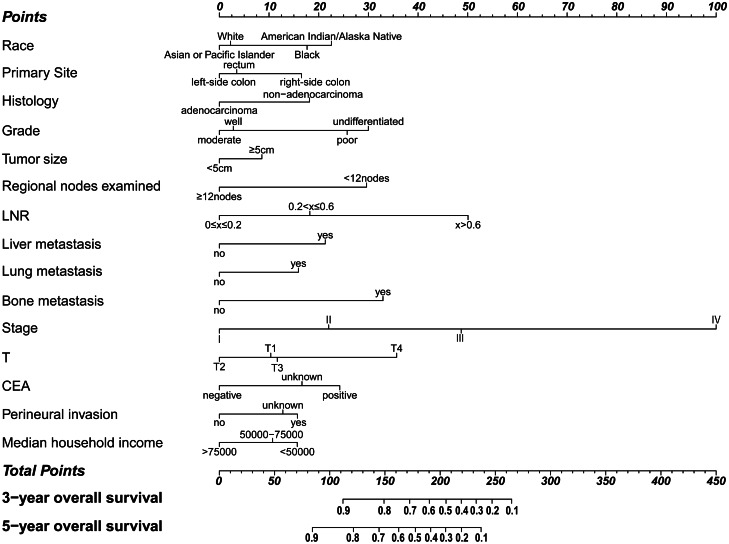
Nomogram for predicting 3- and 5-year OS of early-onset colorectal cancer patients. LNR, lymph node ratio; CEA, carcinoembryonic antigen

A prognostic nomogram to predict 3- and 5-year OS was established, which contained the independent prognostic factors identified from the multivariable Cox regression analysis (Fig. 3). The corresponding score of each variable can be obtained by projecting to the top “points” axis according to the patient’s actual situation. In the same way, the total points are obtained by adding the corresponding scores of each variable. By projecting the total points to the bottom “3-year overall survival” and “5-year overall survival” axis, the 3- and 5-year OS can be estimated.

For instance, a 45-year-old White patient (3 points) with right-sided colon (16 points) adenocarcinoma (0 points), T4 (35 points), without lung, liver, or bone metastasis (0, 0, and 0 points), TNM stage III (48 points), poor differentiated (25 points), tumor size > 5 cm (8 points), examined 12 regional lymph nodes (0 points), LNR > 0.6 (50 points), CEA positive (24 points), without perineural invasion (0 points), median household income 70,000 dollars (10 points) would have a total of 219 points, which means a predicted 3-year OS of 40.0% and predicted 5-year OS of 20.0%.

## Validation of the prognostic nomogram

**Fig. 4:**
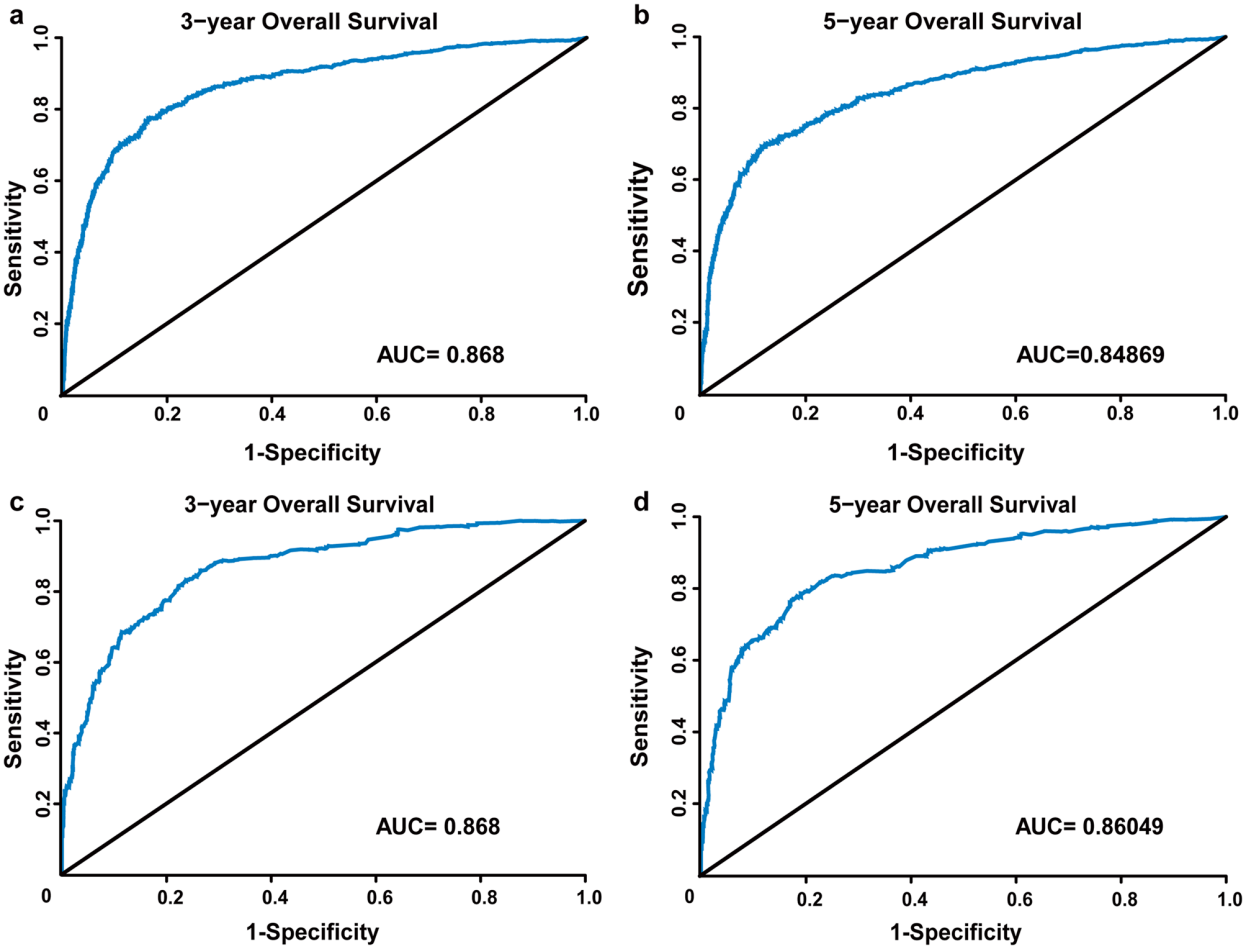
ROC curves and AUC values for training and testing cohort. **a** 3-year OS in the training cohort. **b** 5-year OS in the training cohort. **c** 3-year OS in the testing cohort. **d** 5-year OS in the testing cohort. AUC, area under the ROC curve

To evaluate the discriminatory ability of constructed nomogram, the C-index value and AUC value were applied in this study. The C-index of the nomogram was 0.840 (95% CI 0.827–0.854) and 0.837 (95% CI 0.816–0.857) in the training and testing cohort, respectively. Moreover, the 3- and 5-year AUC values of the nomogram were 0.868 and 0.84869, respectively, in the training cohort, corresponding to 0.868 and 0.86049 in the testing cohort (Fig. 4). Thus, both the C-index and the 3- and 5-year AUC values of the nomogram were over 0.75 and more close to value 1.0, which suggested that the constructed nomogram in our study has good discriminatory ability for OS prediction.

**Fig. 5:**
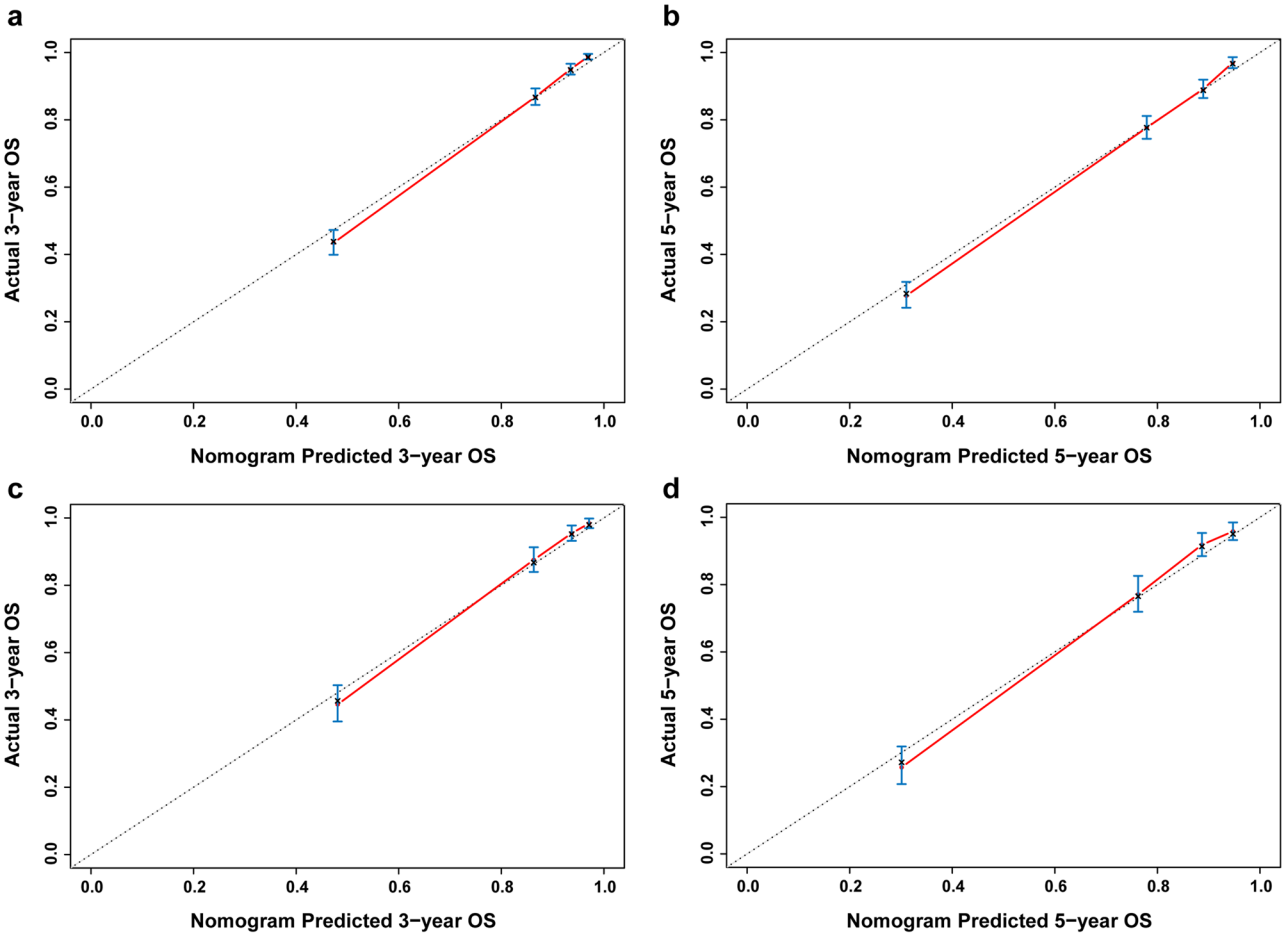
Calibration plots of the nomogram for predicting 3- and 5-year OS in the training cohort (**a**, **b**) and testing cohort (**c**, **d**) respectively. The actual OS is plotted on the y-axis; the nomogram-predicted OS is plotted on the x-axis. OS, overall survival

The calibration of our nomogram was assessed by calibration plots. Actual OS was plotted on the y-axis, and nomogram-predicted OS was plotted on the x-axis. The calibration plots of the established nomogram displayed bare deviations from the 45-degree diagonal reference line both in training cohort and testing cohort **(**Fig. 5**)**, which indicated optimal agreement between the actual observed survival and the nomogram-predicted survival.

### Comparison of nomogram with TNM stages

Moreover, we compared the prediction ability of the nomogram and the TNM staging system. Compared with the C-index of the constructed nomogram (0.840, 95% CI 0.827–0.850), the C-index of the TNM staging system was lower (0.804, 95% CI 0.788–0.820, *P* < 0.001). More importantly, the constructed nomogram yielded a larger log-likelihood and a smaller AIC value than the TNM stage (Table 3). All the above results implied the stronger predictive power of the nomogram than the TNM staging system. And the same result was also observed in the testing cohort.

**Table. 3:** Comparison of nomogram with the TNM staging system

	Nomogram		TNM stage		*P*-value
Training cohort					
C-index (95% CI)	0.840 (0.827–0.850)		0.804 (0.788–0.820)		*P* < 0.001
Likelihood	− 5322.6		− 5462.3		*P* < 0.001
AIC	10,701.2		10,936.6		/
Testing cohort					
C-index (95% CI)	0.837 (0.817–0.858)		0.801 (0.778–0.825)		*P* < 0.001
Likelihood	− 2104.4		− 2159.8		*P* < 0.001
AIC	4264.9		4331.6		/

*C-index* concordance index, *95% CI* 95% confident interval, *AIC* Akaike information criterion

## Discussion

In contrast to the decreasing incidence in older populations, the incidence of EO-CRC patients had increased since the mid-1990s. Accurate survival prediction for EO-CRC patients is important in informing the accurate prognosis of patients and in making personal clinical decisions. Many prognostic factors affecting long-term survival were not sufficiently utilized. Currently, the optimal method for predicting the survival outcome of EO-CRC patients is unclear. Based on large population and multi-institution data from the SEER database, the present study used independent clinicopathological and socio-economic factors to establish and internally validate a nomogram for predicting the 3- and 5-year OS of individual EO-CRC patients.

This study is essential because the nomogram can represent complex mathematical formulas with intuitive visualization results and quickly estimate clinical outcomes without complicated calculations, facilitating individual prognosis prediction and clinical decision-making regarding the treatment and surveillance [18]. Besides, the data of this study were extracted from the openly accessed SEER database, which ensures the sample size sufficient.

Another strength of this study is reflected in the fact that it involved dozens of clinicopathological and socio-economic variables which were associated with the prognosis of EO-CRC in previous reports. Survival outcome is different in colorectal patients with varied primary tumor location. Several previous studies, including meta-analyses, demonstrated that patients with the left-sided disease were significantly associated with a better overall survival rate than those with the right-sided disease [19–21], which is consistent with the present study (HR = 0.72, 95% CI 0.61–0.86, *P* < 0.01). Moreover, our result showed that rectal cancer was higher than right-side colon cancer in terms of OS (HR = 0.76, 95% CI 0.62–0.95, *P* = 0.015), which is in accordance with the previous study [22]. Based on our multivariate analysis, tumor size was also an independent factor for improved OS (HR 1.17, 95% CI 1.01–1.37, *P* = 0.038), which was in agreement with previous reports [23–26]. Previous researches have revealed that a high lymph node ratio (LNR) was significantly correlated with inferior overall and disease-free survival in stage III [27–29] and stage IV [30–32] colorectal cancer patients, which is in line with this study.

For cancer patients, socio-economic status (SES) was reported to be a significant predictor of prognosis [33], which was not considered in most previous nomograms [9–14]. Previous studies showed that patients with low SES resulted in a worse prognosis than those with high SES [34–36]. Similarly, in our study, we also identified the significant association between survival outcome and median household income, a measure indicator of SES. As for the low SES population, they less frequently participate in cancer screening programs, resulting in an advanced stage CRC diagnosis while not at an early stage [37]. Moreover, the worse access to health services and high-quality treatments accelerates their bad outcome [37].

Furthermore, the performance of the constructed nomogram was comprehensively evaluated. Firstly, the constructed nomogram showed good discriminatory ability, with a high C-statistic of 0.840 and the 3- and 5-year AUC values of 0.868 and 0.84869 respectively. What’s more, the calibration plots for 3- and 5-year OS probabilities showed barely any deviations from the reference line (Fig. 4), which means the nomogram-predicted survival would be similar to the actual observed survival. Moreover, the same results were also confirmed in the testing cohort, which further implies the strong predictive ability of our nomogram model. Most importantly, compared with the TNM staging system, the nomogram displayed better predictive activity with a higher C-index (0.840 vs 0.804, *P* < 0.001), larger log-likelihood, and smaller AIC value. The results above collectively suggested that the established nomogram might be utilized as a more powerful and conventional tool to predict survival outcomes for patients with EO-CRC.

Our study shows more strength than previous related nomograms. On the one hand, unlike previous nomograms that just included patients with colon cancer [38–40], our nomogram focused on patients with colon cancer and those with rectal cancer, no matter the stage situation. On the other hand, our nomogram involved some distinct variables, such as SES and LNR, which were also reported to be important predictors of prognosis. And our research is the only study including the socio-economic status of patients in the nomogram.

Of note, the present study had some limitations. Firstly, several vital prognostic factors, such as KRAS, BRAF, microsatellite instability (MSI), tumor regression grade, circumferential resection margin (CRM), were inaccessible in the SEER database, thus did not incorporate in the proposed nomogram. Secondly, the nomogram was devoid of treatment information like surgical procedures and chemotherapy regimens, which greatly affected survival outcomes. Thirdly, although the information of some factors, such as LNR and perineural invasion, may restrict the application of constructed nomogram preoperatively, the nomogram indeed shows a solid ability to predict postoperative patients’ overall survival. In addition, the selection bias could not be ignored because of the retrospective nature of the study. Besides, the constructed nomogram includes relatively more variables, so it requires a high degree of integrity of relevant information, probably affecting the practicability. Last, this study did not involve any external validation based on other populations. Therefore, it is unclear whether the nomogram can be directly applied to other populations, and its universality needs further verification and prospective evaluation.

## Conclusion

A novel nomogram for EO-CRC patients based on independent clinicopathological and socio-economic variables was developed and internally validated, which is superior to the TNM staging system. In addition, the nomogram could facilitate postoperative individual prognosis prediction and clinical decision-making.

## Data Availability

The data and materials are available on reasonable request from the corresponding author.
